# Detection of Hypervirulence Genes and Drug Resistance in *Klebsiella pneumoniae* in Diagnostic Microbiology

**DOI:** 10.1155/sci5/8545710

**Published:** 2025-08-03

**Authors:** Shamitha S. Rao, Anisha M. Fernandes

**Affiliations:** Department of Microbiology, Kasturba Medical College Mangalore, Manipal Academy of Higher Education, Manipal, India

**Keywords:** aerobactin, antimicrobial resistance, biomarkers, classical *Klebsiella pneumoniae*, hypervirulent *Klebsiella pneumoniae*, mucoid phenotype

## Abstract

**Objective:** Hypervirulent *Klebsiella pneumoniae* (hvKp) is emerging and gaining notoriety due to the acquisition of drug resistance. Differentiation of hvKp from classical *Klebsiella pneumoniae* (cKp) is essential for prompt initiation of therapy to prevent metastasis, detection of occult abscesses, and site-specific management for better patient outcomes.

**Methods:** A total of 300 *K. pneumoniae* isolates from various clinical specimens were collected from 256 patients to determine their clinical profiles, antibiograms, risk factors, and patient outcomes. Hypermucoviscosity was demonstrated via a phenotypic string test. The hvKp pathotype was classified by molecular detection of the virulence genes *rmpA* and/or aerobactin-*iucA*.

**Results:** *K. pneumoniae* infections affected the older age group (> 50 years) of both sexes, with a male preponderance (62.89%). Urinary tract infections were the most common clinical presentation (37.33%). Among the 300 isolates, 17 (5.66%) possessed hypervirulence genes, and 281 (93.66%) isolates were string test positive. Pyogenic liver abscess was more frequently observed in hvKp infections (5.88%) than in cKp infections (1.76%). Multiple sites were involved in 35.29% of the hvKp infections. (*p* < 0.05). Hypertension was the common comorbidity observed in the majority of the 256 patients (61.32%). The ICU stay (64.70%) predisposed patients to hvKp infections (*p* < 0.05). Compared with hvKp, cKp presented high rates of resistance to antibiotics. Although extended-spectrum beta-lactamase (ESBL) producers were significantly more common in cKp, 41.1% of hvKp strains were ESBLs. Carbapenem resistance and multidrug resistance were observed in 35.29% of the hvKp strains. The mortality rate in patients infected with hvKp was 23.52%.

**Conclusion:** The potential for occult abscess and metastasis with life-threatening complications necessitates prompt, accurate identification of hvKp. Convergence of hvKp and cKp with shared traits poses a diagnostic and therapeutic dilemma for clinicians. The combination of genetic markers such as *rmpA* with *iucA* has high reported diagnostic accuracy. Further studies are needed to better characterize hvKp in the clinical laboratory.

## 1. Introduction

*Klebsiella pneumoniae* (Kp) is an opportunistic Gram-negative pathogen that causes community-acquired and nosocomial infections, affecting various organs, often leading to life-threatening illness [1]. Its ability to acquire mobile genetic elements has facilitated the spread of drug resistance among strains.

There are two main pathotypes: hypervirulent *Klebsiella pneumoniae* (hvKp) and classical *Klebsiella pneumoniae* (cKp). cKp is commonly associated with nosocomial infections and multidrug resistance. It frequently affects older individuals with underlying disease and involves a single site [2].

Currently, a second hvKp pathotype is emerging, which is distinct from the classical strain [2]. HvKp is more virulent than cKp. These strains are generally community-acquired [3, 4], affect all age groups, and have the capacity to cause multisite infections in young, healthy individuals with no history of illness [2, 5]. Initial hvKp isolates presented low rates of resistance [2].

The development of multidrug-resistant (MDR) strains is a growing concern. Extended-spectrum beta-lactamase (ESBL) and carbapenem resistance (CR) are increasingly prevalent among *K. pneumoniae* clinical isolates. Through the transfer of genetic material, hvKp gains antimicrobial resistance from cKp; conversely, cKp acquires virulence factors from hvKp, causing diseases even in healthy individuals [5–7]. As a result of this evolution, MDR and extensive drug-resistant (XDR) hvKp strains have been reported [2, 5]. These strains represent the ultimate “superbug,” possessing both a hypervirulent pathotype and antimicrobial resistance. In 2024, the World Health Organization (WHO), in its Global Antimicrobial Resistance and Surveillance System on Emerging Antimicrobial Resistance Reporting (GLASS-EAR), reported increased global CR among hvKp strains [8].

This poses new challenges for clinicians in terms of differentiation and management. Hypermucovisocity, demonstrated by the string test, was initially used as a marker for hypervirulence; however, studies have shown that this association is not consistent [1]. The present focus is to distinguish between the two pathotypes via biomarkers. The mucoid regulator genes (*rmpA* and *rmpA2*) and quantitative siderophore synthesis genes (a dominant siderophore, aerobactin, encoded by the *iucA* gene) [2] have been recently used to identify hvKp [6].

In India, studies on the recent emergence of convergent strains of *K. pneumoniae* are rare [7, 9, 10], necessitating further investigations to facilitate prompt and effective patient management. In light of this looming global threat, the WHO in 2024 urged systematic surveillance and strengthening of clinical laboratories for the detection of hvKp [8]. The current study focused on the detection and association of hypervirulence genes with clinical profiles, mucoid phenotypes, antibiograms, and patient outcomes [11].

## 2. Materials and Methods

An in vitro prospective study was carried out on samples received from inpatients (IP) visiting two tertiary care hospitals affiliated with our center. Simple random sampling was performed to collect a total of 300 clinically significant nonrepetitive *K. pneumoniae* isolates from Asian patients aged > 1 year. The sample size was calculated based on a recent study [6], which reported an incidence of 13.9% of hvKp infections, with 5% relative precision and a 95% confidence level. The *K. pneumoniae* isolates identified by the VITEK 2 Compact (Biomerieux) system from blood; sterile body fluids; urine, pus, and exudates; and tissue and respiratory samples sent for culture and sensitivity by clinicians, based on clinical suspicion of specific organ infections, were included. Sample collection was performed according to standard microbiological sampling procedures. Samples were screened by microscopy, and acceptability was determined prior to culture.

The isolate was considered clinically significant if it fulfilled the following criteria [7]:

1. Urine: Colony count of > 10^5^ colony forming units/mL with moderate to many pus cells
2. Blood and sterile fluids: Any microorganisms found at the sterile site were considered significant.
3. Pus, exudate, and tissue: presence of 8–10 pus cells/low-power field.
4. Sputum: As per the Murray Washington criteria, a specimen was accepted if it contained < 10 squamous epithelial cells/LPF and > 25 white blood cells/LPF.
5. Bronchoalveolar lavage: 10^4^–10^5^ colony forming units/mL.
6. Endotracheal aspirate: > 10^5^ colony forming units/mL and > 5 polymorphonuclear cells/high-power field.

Following Institutional Ethics Committee clearance, *K. pneumoniae* isolates collected over a period of 8 months (June 2023–January 2024) were stored in 20% glycerol broth at −20°C until use. *K. pneumoniae* isolated from different samples of a single patient or from the same site with different sensitivity patterns was considered to be a different isolate.

Clinical information (sample accession number, age, sex, underlying disorders, single- or multiple-site infection, monomicrobial or polymicrobial etiologies, and clinical outcomes) was collected from the Medical Records Department. The antibiograms of *K. pneumoniae* isolates, including ESBL, CR, and MDR strains, were collected from laboratory reports.

A string test was performed to determine if the isolates presented a hypermucoviscous phenotype. The *K. pneumoniae* isolates were subsequently grown on MacConkey agar and incubated overnight at 37°C. A test was defined as positive if it yielded a viscous string of > 5 mm when the inoculation loop was pulled away from an isolated colony [2, 12, 13].

### 2.1. Antibiotic Susceptibility Test

The results of conventional Kirby–Bauer disk diffusion and MICs from the automated VITEK 2 Compact (Biomerieux) system were interpreted via the Clinical and Laboratory Standards Institute (CLSI) 2023 [14].

The antibiotics included amoxicillin/clavulanic acid, cotrimoxazole, ciprofloxacin, gentamicin, amikacin, piperacillin/tazobactam, cefoperazone/sulbactam, imipenem, meropenem, ceftriaxone, and cefepime. Susceptibility to nitrofurantoin and norfloxacin was tested in the urine isolates.

ESBLs were identified using the VITEK 2 Compact (Biomerieux) system Advanced Expert System Library Report. An isolate was considered carbapenem-resistant if resistance to imipenem or meropenem was detected [6, 14]. Resistance to three or more classes of drugs was considered MDR [6].

### 2.2. Molecular Testing

DNA extraction from the *K. pneumoniae* isolates was performed via the boiling method [15]. The isolates stored in 20% glycerol broth were streaked onto blood agar and incubated overnight at 37°C to obtain pure and isolated colonies. The bacterial colonies were then emulsified in sterile Eppendorf tubes with 150 μL of nuclease-free water to form a milky white suspension. The suspension was vortexed and placed in a dry bath at 98°C for 10 min with intermittent vortexing to lyse the cells. The lysate was centrifuged at 13,000 rpm for 5 min to separate the pellet and supernatant. One hundred microliters of the supernatant containing the crude DNA was aliquoted into another sterile Eppendorf tube and stored at −20°C.

The *iucA* (aerobactin) and *rmpA* (mucoid phenotype) HPLC primers were procured from EUROFINS Genomics India Pvt. Conventional multiplex PCR was performed via a PCR amplification kit (TaKaRa Taq) as described previously [15]. The primers used in the study are listed in Table 1. The reaction mixture contained a total volume of 25 μL, including 1 μL of DNA, 250 mM dNTPs, 0.1 μM primers, 1x polymerase buffer, 2.5 U of Taq DNA polymerase, and the remaining volume with nuclease-free water. The cycling conditions are listed in Table 2.

The amplification products were detected via a gel electrophoresis system at 120 V and 200 mA current. The amplicons with gel loading dye and a DNA ladder of 100 bp for reference were added to wells in the 2% agarose gel. The gel was run for 60 min, after which it was stained in 0.05 mg/L ethidium bromide solution for 30 min. The stained gel was then viewed under an ultraviolet transilluminator (Bio-Rad GelDoc Go Imaging System).

All isolates found to be positive by multiplex PCR were subjected to Uniplex PCR for the individual genes. The primers and cycling conditions were the same as those for the multiplex PCR.

CKp or hvKp were classified based on the detection of the *iucA* and *rmpA* genes [16]. If both genes were absent, the isolate was labeled cKp. The presence of either *rmpA* or *iucA* or both genes was recorded as hvKp.

### 2.3. Statistical Analysis

The data collected from medical records were entered into MS Excel, analyzed, presented in the form of tables, and interpreted via JAMOVI Software version 2.4.14. For categorical variables, a chi-square test was performed, and a *p* value < 0.05 was considered to indicate statistical significance. The specificity, positive predictive value (PPV), and accuracy of the string test were calculated via the following formula and are expressed as percentages:

(1) $$\begin{array}{c} \text{Specificity}=\frac{\text{true negatives}}{\left(\text{true negatives}+\text{true positives}\right)},\\ \text{PPV}=\frac{\text{true positives}}{\left(\text{true positive}+\text{false positive}\right)},\\ \text{Accuracy}=\frac{\text{true positives}+\text{true negatives}}{\left(\text{true positives}+\text{false positives}+\text{true negatives}+\text{false negatives}\right)}. \end{array}$$

## 3. Results

**Figure 1:**
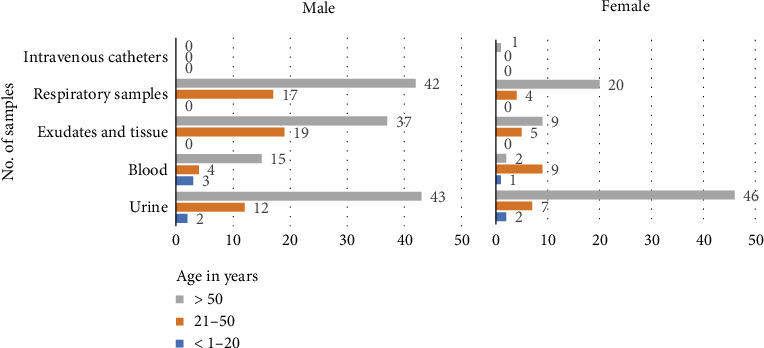
Age and sex distributions of *Klebsiella pneumoniae* isolates from different samples.

A total of 300 *K. pneumoniae* isolates were obtained from various clinical specimens collected from 256 patients. A greater number of samples were collected from males (161/256, 62.89%) than from females (95/256, 37.10%). Most participants (188/256, 73.43%) were aged above 50 years, with 23.43% (60/256) and 3.12% (8/256) falling under the age groups of 21–30 and below 20 years, respectively. The mean age was 59 years for both males and females (Figure 1).

Isolates from different clinical samples in decreasing order of frequency were urinary tract infections (112/300; 37.33%) > respiratory samples (83/300; 27.66%) > exudate and tissue (70/300; 23.33%) > blood (34/300; 11.33%) > intravenous catheters (1/300; 0.33%).

In females, the most common clinical presentation was UTI (55/95; 57.89%), and in males, respiratory tract infections (59/161; 36.64%) affected individuals above 50 years of age (Figure 1, Table 3).

### 3.1. Detection of Virulence Factors: hvKp v/s cKp

**Figure 2:**
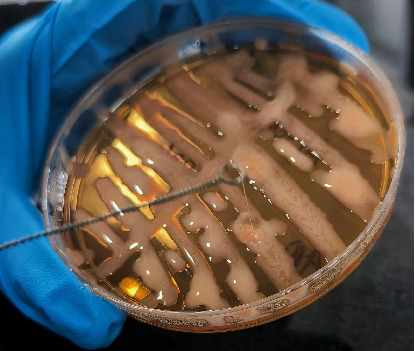
Demonstration of hypermucoviscosity via a positive phenotypic string test.

Hypermucoviscosity was demonstrated via the phenotypic string test, and the virulence markers *rmpA* and *iucA* were detected via genotypic methods. Differentiation of classical (cKp) and hypervirulent (hvKp) *K. pneumoniae* was based on the presence of the *rmpA* and/or *iucA* genes. Among the 300 isolates tested, 281 (93.66%) were string test positive (Figure 2).

**Figure 3:**
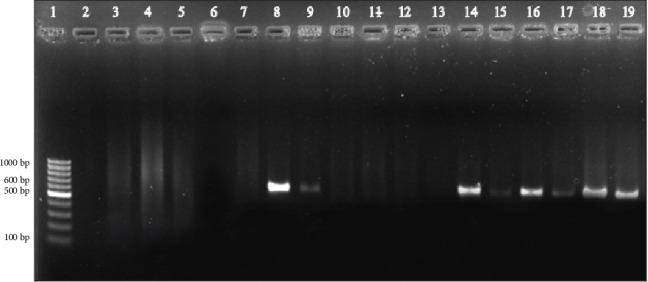
Gel image of the amplified PCR products of the *iucA* and *rmpA* genes in the *Klebsiella pneumoniae* isolates included in the study. LANE 1, DNA ladder, LANE 2, negative control (NC), LANE 3-7, negative samples, LANE 8, *iucA*, LANE 9, *rmpA*, LANE 10-13, negative samples, LANE 14, *iucA*, LANE 15, *rmpA*, LANE 16, *iucA*, LANE 17, *rmpA*, LANE 18, known positive control (KPC) of *iucA*, and LANE 19, KPC of *rmpA*.

Among the 300 isolates, 17 (5.66%) were positive for the following virulence genes: 15/17 (88.23%) were positive for the *iucA* gene, and 11/17 (64.70%) were positive for the *rmpA* gene. A total of 9/17 (52.94%) isolates were positive for both the *iucA* and the *rmpA* genes (Figure 3). These 17 isolates were classified as hvKp. All 17 isolates were obtained from different patients.

All 17 hvKp isolates tested positive in the string test, whereas 93.28% of the cKp isolates (264/283) tested positive in the string test (Table 3).

### 3.2. Patient Demographics and Clinical Profile of hvKp

Among the 17 hvKp isolates, 11 samples were collected from males (64.70%) and 6 were collected from females (35.29%). Most participants (13/17, 76.47%) were aged above 50 years, with 23.52% (4/17) under the age group of 21–30 years and no patients younger than 20 years. The mean age was 57.8 years in males and 59.7 years in females.

In decreasing order of frequency, hvKp isolates were obtained from exudate and tissue (8; 47.05%), respiratory samples (5; 29.41%), urine and blood (11.76% of each), and nil isolates from intravenous catheters. The clinical presentation of pyogenic liver abscess was greater in hvKp (1/17, 5.88%) than in cKp (5/283, 1.76%) Table 3.

In our study, we observed multiple-site infections in 11.71% of 300 cases; 35.29% of hvKp (6/17) and 8.48% of cKp (24/283) infections were associated with multisite infection (*p* = 0.018).

Among the two groups, polymicrobial infections were relatively greater in hvKp (4/17; 23.52%) than in cKp (47/283; 16.60%), although this difference was not statistically significant.

### 3.3. Risk Factors for cKp and hvKp

Among the 256 patients, 179 (69.92%) had comorbidities. Hypertension was the most common comorbidity in many patients (157/256; 61.32%), followed by diabetes mellitus (135/256; 52.73%).

Indwelling devices were a predisposing factor of cKp, which was significantly greater in our study (130/256; 50.78%), with *p* < 0.05. These included central intravenous catheters, urinary catheters, endotracheal tubes, and gastrostomy tubes.

**Figure 4:**
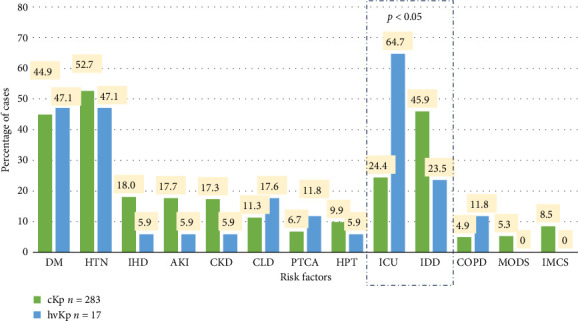
Comparison of risk factors associated with classical (cKp) and hypervirulent *Klebsiella pneumoniae* (hvKp) infections. DM, diabetes mellitus; HTN, hypertension; IHD, ischemic heart disease; AKI, acute kidney injury; CKD, chronic kidney disease; CLD, chronic liver disease; PTCA, percutaneous transluminal coronary angioplasty; HPT, hyperthyroidism; ICU, intensive care unit; IDD, indwelling devices; COPD, chronic obstructive pulmonary disease; MODS, multiple organ dysfunction syndrome; IMCS, immunocompromised state; statistical test: chi-square test.

All patients in the hvKp group had comorbidities. Hypertension and diabetes mellitus were equally common (8/17; 47.05%), followed by chronic liver disease (3/17; 17.64%) and catheterized patients (4/17; 23.52%) (Figure 4).

The number of MODS and immunocompromised states was greater in the cKp group than in the hvKp group (5.85% v/s 0% and 9.37% v/s 0%, respectively). The ICU stay (11/17, 64.70% vs. 24.38% in cKp) (*p* < 0.05) was found to predispose patients to hvKp infections.

Chronic liver disease and chronic obstructive pulmonary disorder (COPD) were observed in greater proportions of hvKp patients than cKp patients (17.64% and 11.76% vs. 11.30% and 4.95%, respectively).

### 3.4. Resistance Profiles of cKp and hvKp

**Figure 5:**
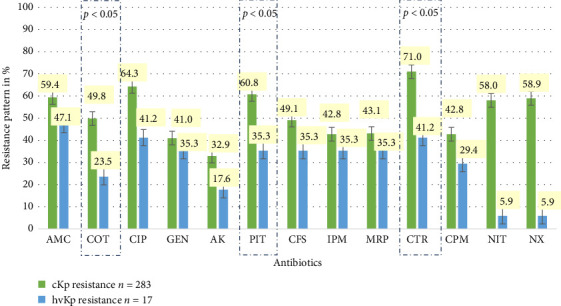
Comparison of antibiotic susceptibility patterns of classical *Klebsiella pneumoniae* (cKp) and hypervirulent *Klebsiella pneumoniae* (hvKp). AMC, amoxycillin/clavulanic; COT, cotrimoxazole; CIP, ciprofloxacin; GEN, gentamicin; AK, amikacin; PIT, piperacillin/tazobactam; CFS, cefaperazone/sulbactam; IPM, imipenem; MRP, meropenem; CTR, ceftriaxone; CPM, cefepime; NIT, nitrofurantoin; NX, norfloxacin. Statistical test: chi-square test.

**Figure 6:**
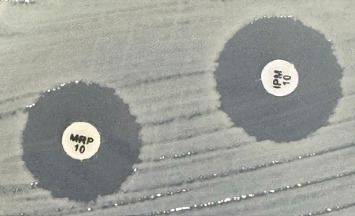
Zones of inhibition of a *Klebsiella pneumoniae* isolate to imipenem and meropenem by the conventional Kirby–Bauer disk diffusion method.

Compared with the hvKp group, the CKp group presented high rates of resistance to common antibiotics, as shown in Figure 5. Resistance to cotrimoxazole, piperacillin/tazobactam, and ceftriaxone among the cKp isolates was significantly greater (*p* < 0.05).  Figure 6 shows the zones of inhibition to imipenem and meropenem according to the Kirby–Bauer Disk diffusion test.

Among the 300 isolates, the rates of MDR were 57.33% and 58.65% and 35.29% for cKp and hvKp, respectively.

Similarly, 70% of the 300 isolates were ESBLs, among which rates were significantly greater in the cKp group than in the hvKp group (71.73% v/s 41.17%) (*p* = 0.008) (Table 4).

The prevalence of CR in hvKp was 6/17 (35.29%), and that in cKp was 128/283 (45.22%).

### 3.5. Outcome

The mortality rate in patients infected with hvKp (4/17; 23.52%) was higher than that in patients infected with cKp (45/239; 18.82%). However, this difference was not statistically significant.

## 4. Discussion

There is no established definition to distinguish hvKp from cKp [9]. Several markers associated with hypervirulence are expressed at greater levels in hvKp than in cKp. Initially, hypermucoviscosity was demonstrated via the phenotypic “string test” [1, 2]. Russo et al. [2] identified low accuracy, sensitivity, and specificity for hvKp detection via the string test in comparison with various virulence plasmid biomarkers. In our study, we observed overall string test positivity in 93.66% (281/300) of the isolates. All 17 gene-positive isolates defined as hvKp were string test positive; however, 264 of the 283 cKp isolates (93.28%) were also string test positive. We observed a low specificity, PPV, and accuracy of the string test, with values of 6.71%, 6.05%, and 12%, respectively.

Capsular production is a key mechanism for the survival of *K. pneumoniae*, allowing evasion of phagocytosis, antimicrobial peptides, complement, and antibodies. Serotyping on the basis of capsular production has identified nearly 80 distinct types, with K1 and K2 being the most frequently expressed in hvKp. A demonstration of capsular serotype-specific genes would provide strong supportive evidence of hypervirulence. Capsular overproduction is controlled by several genes: *magA* (mucoviscosity gene), *rmpA* and *rmpA2* (regulators of the mucoid phenotype), and peg-344 (metabolic transporter). Among these genes, *rmpA* is a major contributor to the pathogenicity of hvKp due to abundant capsule production. While the hypermucoviscosity (hmv) phenotype is associated with increased production of capsular polysaccharides, it is dependent on other cellular factors. Hence, the hmv phenotype demonstrated by a positive string test may be observed without the expression of capsular genes [17].

Previous studies have shown that hvKp strains can produce multiple types of siderophores, which increase iron uptake and bacterial growth [4, 10, 18]. Among these, aerobactin and salmochelin are hvKp specific, whereas yersiniabactin (*ybt*) and enterobactin (*ent*) are frequently produced by both cKp and hvKp. More than 90% of hvKp isolates have been shown to produce aerobactin, making it a good marker for hypervirulence. High expression of salmochelin (*iroA* locus gene) is also observed in hvKp. A high combined occurrence of the aerobactin and salmochelin genes in hvKp was reported in a study of over 2500 *K. pneumoniae* genomes [17].

The combination of hypermucoviscous factors and iron acquisition systems is a well-defined marker for the identification of hvKp [2, 3]. Various studies have suggested the detection of the virulence genes *rmpA* and/or *rmpA2* in combination with *iucA*, *iroB*, or *peg-344*. A combination of these markers has high diagnostic accuracy in identifying hvKp [2, 16]. Ventura et al. [19] suggested that concurrent detection of all 5 of the above markers would more conclusively differentiate classical and hypervirulent strains. Owing to financial constraints, our research could not encompass the detection of multiple molecular markers, which would have made our study more robust. Our study employs the combined detection of two virulence genes, the regulator of mucoid phenotype (*rmpA*) and the iron siderophore aerobactin synthetase gene (*iucA*), via molecular methods to distinguish hvKp from cKp. We concur with Russo et al. that these two biomarkers are superior to culture-based phenotypic methods in the detection of hvKp isolates. Among the 17 isolates identified as hvKp, 15 produced the siderophore aerobactin *iucA,* and 11 produced *rmpA*. Both genes were detected in 9/17 (52.94%) isolates, indicating that a combination of *iucA* and *rmpA* was an appropriate diagnostic marker of hvKp.

Parrott et al. [12] reported that all infections with *K. pneumoniae* had a strong male predominance and affected older adults. In our study, we noted that the highest number of *K. pneumoniae*–associated infections affected an average age of 59 years in both sexes, with the highest number of cases in males (62.89%) compared with females (37.10%).

The majority of *K. pneumoniae* in our study were isolated from cases of UTI (37.33%), followed by respiratory samples (27.66%). A prior study [6] performed at the same center revealed a similar clinical preponderance of UTIs (34.88%), followed by septicemia (27.13%). In other studies [4, 10, 12, 20], isolates were predominantly obtained from bloodstream infections; however, we reported that only 11.33% of the isolates were obtained from blood. The higher rates of infections in males could be attributed to social bias, as a greater proportion of patients visiting the hospital are male. However, a greater proportion of UTIs affect females, which can be attributed primarily to anatomical differences. Among our 300 isolates, one strain was obtained from the culture of an indwelling catheter. At our center, we discourage the culture of tips and catheters, as growth commonly represents colonizers. Intravenous catheter tips are accepted if accompanied by a blood culture. This single isolate grown in both blood and tip cultures was identified as cKp. However, these single data points do not allow for meaningful statistical comparisons.

Hepatic abscess in the absence of biliary tract disease is a hallmark clinical presentation of hvKp [2, 8]. Several studies in the Asian-Pacific region [13, 15, 21] have shown that hvKp is strongly associated with liver abscess. We observed that hvKp caused pyogenic liver abscess in 5.88% of hvKp infections compared with 1.76% of cKp infections. Wang et al. [13] reported that the expression of the *rmpA* and *iucA* genes facilitates invasion of the liver, leading to liver abscesses, often with multiple foci.

The ability of hvKp to metastasize has been reported to cause life-threatening infections [12, 22, 23], especially in patients with diabetes [24]. In our study, multiple-site infections were observed in 35.29% of the hvKp strains, which was statistically significant. Polymicrobial infection has not been previously identified in association with hvKp [25]; however, we noted polymicrobial infections in 23.52% of hvKp-related illnesses.

Important risk factors for hvKp infections, which were previously identified, include community-acquired infections, solid malignancies, and diabetes mellitus [1, 26]. Others reported that patients with indwelling devices and ICU admission are at increased risk for ESBL-hvKp [26]. In the present study, we identified ICU stay as an independent risk factor for hvKp. Surprisingly, indwelling catheters were an important risk factor for cKp infections. Chronic liver disease was observed in a greater proportion of hvKp isolates than cKp isolates (28/29; 97% v/s 44/59; 75%) in a study by Li et al. [27]. Similarly, chronic liver disease was more frequently associated with hvKp isolates than with cKp isolates (17.64% v/s 11.30%). Previous investigations [25] have suggested that immunosuppression is an independent risk factor for hvKp. Interestingly, none of our patients with hvKp infection were immunosuppressed. Preexisting respiratory tract diseases such as COPD are reported to predispose patients to hvKp, and a study performed at our center [6] reported COPD in 50% of cases. In the present study, COPD was observed in only 6.25% of the patients.

Initial isolates of hvKp presented lower antibiotic resistance rates than cKp strains did; however, there are increasing reports of drug resistance in hvKp strains globally [2, 9, 28]. The emergence of drug-resistant hvKp strains is due to the acquisition of mobile genetic elements that carry resistance determinants. ESBL-producing hvKp was observed in 7.4% of the isolates in a study performed in southern China in 2018 [29]. Studies in the same geographical area [14, 30] revealed the emergence of carbapenem-resistant hvKp from 2023 to 2024. Li et al. [16] reported that 81.5% of hvKp isolates were carbapenem-resistant. In Italy, a study reported 70% MDR hvKp [31]. Data from the Middle East revealed varying incidences of XDR among hvKp (35.7%–84.74%) [32].

Notably, in our study, the hvKp strains were significantly more susceptible to cotrimoxazole, ceftriaxone, and piperacillin/tazobactam than the cKp isolates were. Among the 210 ESBL isolates in our study, 96.66% (203 isolates) were cKp strains. This statistically significant association of ESBLs with the classical pathotype was also reported by Li et al. [29]. Among the hvKp isolates in our study, 41.17% were ESBLs, 35.29% were carbapenem-resistant, and 35.29% were MDR. A study performed at our center in 2021 [6] reported similar rates of ESBLs and MDR bacteria (44.44%). This growing trend of drug resistance reflects the global pattern seen in recent years. Drug-resistant hvKp can spread readily in clinical settings, causing fatal outbreaks [24].

HvKp can cause various systemic infections with high mortality rates [2, 3]. Mortality rates among hvKp and cKp in our study were 23.52% and 18.82%, respectively, which was not statistically significant. Management of hvKp is challenging and requires prompt initiation of therapy to avoid metastasis, detection of occult abscesses, and site-specific management. The emergence of drug resistance among hvKp has complicated its further management. Additionally, cKp strains have been reported to acquire hvKp-specific virulence determinants [2, 9]. The overlap in drug resistance profiles and epidemiology has blurred the distinction between classical and hypervirulent pathotypes. This necessitates more studies to obtain a clear definition and rapid and accurate detection of hvKp for effective management.

## 5. Conclusion

The prevalence of hvKp is increasing due to the emergence of drug resistance. We observed a notable preponderance of hvKp in males and older adults, especially among ICU patients. Pyogenic liver abscess and polymicrobial infections were more common with hvKp. Our study highlights increasing drug resistance with high mortality rates among hvKp. Genetic markers, viz. regulators of the mucoid phenotype (*rmpA*) and the siderophore aerobactin synthetase (*iucA*), are superior to phenotypic methods in the identification of hvKp. The utilization of multiple biomarkers would provide a more conclusive differentiation. Larger cohort studies are needed to develop a clearer definition for hvKp. Early detection and effective management are fundamental for improving patient outcomes, and surveillance aids in preventing outbreaks in health-care settings.

## Figures and Tables

**Table 1:** Forward and reverse primers used for amplification of the target genes *rmpA* and *iucA*.

Gene	Primer sequence	Base pairs
*rmpA* (for)	5′-ACTGGGCTACCTCTGCTTCA-3′	536
*rmpA* (rev)	5′-CTTGCATGAGCCATCTTTCA-3′	

*iucA* (for)	5′-GCATAGGCGGATACGAACAT-3′	556
*iucA* (rev)	5′-CACAGGGCAATTGCTTACCT-3′	

**Table 2:** PCR cycling conditions.

Stage	Temperature (°C)	Time (min)	Cycles
Initial denaturation	95	5	1

Denaturation	95	1	40
Annealing	50	1	
Elongation	72	2	

Final extension	72	7	1

**Table 3:** Differentiation of *Klebsiella pneumoniae* isolates into classical (cKp) and hypervirulent (hvKp) pathotypes.

Sample type	Total no. isolates (%)	Virulence markers					*K. pneumoniae* (%)	
		String test positive (*n* = 281)		Molecular markers				
		cKp (%)	hvKp (%)	*iucA* (%)	*rmpA* (%)	Both *iucA* and *rmpA* (%)	cKp (%)	hvKp (%)
Urine	112 (37.33)	103 (36.65)	2 (11.76)	2	1	1	110 (98.21)	2 (1.78)
Blood	34 (11.33)	29 (10.32)	2 (11.76)	2	0	0	32 (94.11)	2 (5.88)
Exudate and tissue	70 (20.33)	59 (20.99)	8 (47.05)	6	7	5	62 (88.57)	8 (11.42)
Respiratory samples	83 (27.66)	72 (25.62)	5 (29.41)	5	3	3	78 (93.97)	5 (6.02)
Intravenous catheters	1 (0.33)	1 (0.35)	0	0	0	0	1 (100)	0 (0)
Total	300	264 (93.95)	17 (6.04)	15 (5)	11 (3.66)	9 (3)	283 (94.33)	17 (5.66)

**Table 4:** Incidence of ESBL production and MDR in classical (cKp) and hypervirulent *Klebsiella pneumoniae* (hvKp) isolates.

Type of susceptibility	cKp (%) *n* = 283	hvKp (%) *n* = 17	Total (%)	*p* value
MDR	166 (58.65)	6 (35.29)	172 (57.33)	0.0615
NON-MDR	117 (41.34)	11 (64.70)	128 (42.66)	

ESBL	203 (71.73)	7 (41.17)	210 (70)	**0.0085**

*Note:* Statistical test: Chi-square test. *p* value of < 0.05 is statistically significant and has been emphasized in bold.

## Data Availability

The data that support the findings of this study are available from the corresponding author upon reasonable request.
